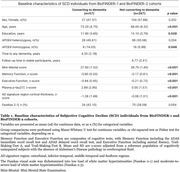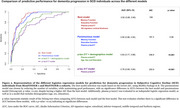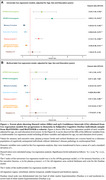# Prediction of progression to dementia in individuals with subjective cognitive decline

**DOI:** 10.1002/alz70856_102807

**Published:** 2025-12-24

**Authors:** María Rivera Sánchez, Sophie E. Mastenbroek, Shorena Janelidze, Pontus Tideman, Niklas Mattsson‐Carlgren, Erik Stomrud, Oskar Hansson, Sebastian Palmqvist, Rik Ossenkoppele

**Affiliations:** ^1^ Clinical Memory Research Unit, Department of Clinical Sciences Malmö, Faculty of Medicine, Lund University, Lund, Sweden; ^2^ Department of Neurology. Marqués de Valdecilla University Hospital, Santander, Cantabria, Spain; ^3^ University of Cantabria, Santander, Cantabria, Spain; ^4^ IDIVAL Health Research Institute, Santander, Cantabria, Spain; ^5^ Amsterdam Neuroscience, Neurodegeneration., Amsterdam, Netherlands; ^6^ Department of Radiology and Nuclear Medicine, Vrije Universiteit Amsterdam, Amsterdam University Medical Center, location VUmc, Amsterdam, Netherlands; ^7^ Memory Clinic, Skåne University Hospital, Malmö, Skåne, Sweden; ^8^ Wallenberg Center for Molecular Medicine, Lund University, Lund, Sweden; ^9^ Clinical Memory Research Unit, Lund University, Malmö, Skåne, Sweden; ^10^ Alzheimer Center Amsterdam, Neurology, Vrije Universiteit Amsterdam, Amsterdam UMC location VUmc, Amsterdam, Netherlands

## Abstract

**Background:**

Identifying Subjective Cognitive Decline (SCD) patients at increased dementia risk is essential for accurate prognosis, and their potential selection for novel disease‐modifying therapies clinical trials, enabling the inclusion of early stages of neurodegenerative diseases. We aimed to identify predictors of progression to dementia in SCD.

**Method:**

We selected SCD individuals from BioFINDER‐1 and BioFINDER‐2 cohorts with at least one follow‐up visit and information available on dementia progression (*n* = 324). Baseline variables evaluated included plasma *p*‐tau217, AD‐signature region cortical thickness, white matter hyperintensities (Fazekas scale), brief executive and memory tests, and *APOE* genotype. Continuous variables were expressed as z‐scores from a cognitively unimpaired reference population without Alzheimer's Disease (AD) pathology. Univariate and multivariable Cox regression analysis, adjusted for age, sex and education, were used to calculate hazard ratios (HRs). Additionally, the area under the receiver operating characteristic curve (AUC) was calculated using logistic regression models combining different variables. The lowest‐AIC (Akaike Information Criterion) model was considered the best. A parsimonious model with similar perfomance was selected by reducing the number of variables. DeLong test was used for AUC comparison between models.

**Result:**

57/324 SCD participants (17.59%) progressed to dementia, including AD (68.42%), vascular (12.28%), Lewy body (10.53%) and other (8,77%) dementias. Mean time to dementia was 4.20 (± 2.18) years (Table‐1). Univariate Cox regression analysis showed a significant association between dementia risk and *APOE4* presence (heterozygosis: HR: 2.54 [1.43, 4.53]; homozygosis: HR: 3.93 [1.74,8.91]), memory (HR: 0.20 [0.10, 0.40]) and executive tests (HR: 0.36 [0.23, 0.57]), plasma *p*‐tau217 (HR: 2.14 [1.74, 2.62]) and ‘AD‐signature’ cortical thickness (HR: 0.48 [0.38, 0.61]), Figure 1A. In multivariable Cox regression analysis, *APOE*4 homozygosis, memory tests, plasma *p*‐tau217 and ‘AD‐signature’ cortical thickness were the strongest predictors for dementia progression (Figure 1B). The best logistic model included memory and executive tests, plasma *p*‐tau217 and ‘AD‐signature’ cortical thickness (AUC 0.92 [0.89, 0.95]). A more parsimonious model excluding cortical thickness had a similar AUC (0.90 [0.86, 0.94]) (Figure 2).

**Conclusion:**

Our findings suggest that an algorithm including plasma *p*‐tau217, *APOE4* genotype, executive and memory tests and ‘AD‐signature’ cortical thickness, could refine SCD individuals’ prognosis in clinical practice and optimize participant selection in preclinical AD trials.